# From implementation to learning: a process-oriented framework to evaluate innovative global health capacity building in fragile and conflict-affected settings

**DOI:** 10.1186/s12960-026-01075-x

**Published:** 2026-05-04

**Authors:** Shadi Saleh, Rim Alaeddine, Tracy Daou, Nisrine El-Hadi, Rania Mansour, Hady Naal

**Affiliations:** 1 Global Health Institute, American University of Beirut, Beirut, Lebanon; 2 Faculty of Health Sciences, American University of Beirut, Beirut, Lebanon; 3 College of Medicine and Sciences, Mayo Clinic, Phoenix, AZ USA

**Keywords:** Framework, Evaluation, Health, Workforce, Capacity building, Fragile and conflict-affected settings

## Abstract

**Background:**

Global Health Capacity Building (GHCB) initiatives are central to strengthening health systems and workforce readiness in Fragile and Conflict-Affected Settings (FCAS). While many evaluation frameworks exist, most were developed for stable contexts and offer limited guidance on how to adapt evaluation processes to environments rife with political instability, limited infrastructure, population mobility, and rapidly shifting conditions. As innovative learning modalities, such as online and blended learning modalities expand in FCAS, there is a critical need for evaluation approaches that are context-responsive, process-oriented, and tailored to fragile settings.

**Method:**

This paper presents the Evaluation of Capacity Building (eCAP) framework, an evidence-informed framework for evaluating GHCB in FCAS. The eCAP framework was developed through a 5-year iterative process by the Global Health Institute at the American University of Beirut, as informed by 3 sequential phases: (1) 3 systematic reviews exploring evaluation methods for GHCB in low- and middle-income countries and in the MENA region; (2) evaluation of 5 case studies implemented in the region; and (3) a synthesis of outcomes and process-related results between 2019 and 2024.

**Results:**

The eCAP framework conceptualizes the evaluation lifecycle in FCAS as a dynamic and adaptive process across three interconnected phases: (1) understanding the program (modality, population, context, level of evaluation, and program logic); (2) implementation (logistics, recruitment, engagement, tool selection, and timing of data collection), and (3) analysis, feedback, and learning. Without prescribing standardized indicators, the framework emphasizes decision-making principles for evaluation that enable adaptation to contextual constraints and other field-based realities. The framework is useful for diverse FCAS, and it has demonstrated feasibility and utility in capturing short-to-medium term outcomes while preserving methodological rigor under challenging and unstable conditions.

**Conclusion:**

The eCAP framework addresses a key gap in the evaluation of GHCB initiatives in FCAS by offering a structured yet adaptable approach grounded in ample field-based evidence. The framework provides practical guidance for researchers, implementers, and funders seeking to design and evaluate capacity building initiatives in complex humanitarian environments. Ultimately, this framework has implications for strengthening evaluation practices, improving programmatic learnings, and guiding policy and funding decisions related to capacity building in FCAS.

## Background

Global Health Capacity Building (GHCB) initiatives play a crucial role in responding to humanitarian emergencies and enhancing population health, development, and research systems in Fragile and Conflict-Affected Settings (FCAS), particularly in the Middle East and North Africa (MENA) region. GHCB initiatives are defined as interventions that aim to enhance local capacities through strengthening skills, knowledge, and systems among health and humanitarian actors. These could take on the form of interventions through mentorship, courses, workshops, field-based practices and others delivered through in-person, online, or blended formats. Ultimately these initiatives contribute to sustainable development within complex environments that often lack stable governance, sufficient resources, and functional systems [1]. FCAS present distinct and compounding challenges, including security threats, sociopolitical instability, economic hardship, and limited infrastructure, alongside data limitations, ethical constraints, and cultural and language barriers [2, 3]. Navigating these realities requires adaptive strategies, context-responsive decision-making, and meaningful community engagement throughout the design and implementation of GHCB initiatives.

Evaluation is a critical component of successful capacity-building programs. It allows practitioners to assess the effectiveness and efficiency of interventions, ensuring alignment with the needs of the population and adapting strategies in response to evolving realities [4]. Evaluation further supports evidence-informed decision-making and resource allocation, ensuring that investments are directed toward initiatives with the greatest potential impact [5, 6]. Beyond accountability and transparency, robust evaluation processes foster continuous learning and innovation, strengthening the sustainability and relevance of GHCB efforts in complex humanitarian environments.

A range of established models has informed the evaluation of capacity building initiatives. Common models include the Kirkpatrick, Logic, RE-AIM, and Community of Inquiry (CoI) frameworks, which provide structured approaches to evaluate initiatives, including clear articulation of program goals, activities, and expected outcomes. The Kirkpatrick model focuses on different levels of training outcomes and provides insights into immediate reactions, learning, behavioral changes, and results [7]. The Logic Model supports visualizing program components and the relationships between them, facilitating clear communication and planning [8]. The RE-AIM framework emphasizes the broader individual-level impact of programs, considering reach, adoption, and sustainability, which are crucial in fragile settings [9]. Additionally, the CoI model emphasizes cognitive, social, and teaching presence particularly within online interactions in settings where remote learning is necessary [10].

Despite their conceptual value, these frameworks often lack the flexibility required to adapt to rapidly changing and unpredictable environments that characterize FCAS, where political instability and ongoing conflicts are common. While effective in structuring evaluation components in theory, these frameworks provide limited guidance on how evaluation processes can be adapted and optimized in practice under conditions of political instability, conflict, and systemic disruption. For instance, the linear progression and structured approach of the Kirkpatrick and Logic models may not fully capture the non-linear pathways through which capacity building outcomes emerge, particularly when external shocks alter implementation trajectories. Additionally, the CoI model’s focus on online learning can be constrained by infrastructural limitations, such as unreliable internet access and power outages. Collectively, these limitations risk undermining the ability of evaluations to capture contextual dynamics, lived experiences, and meaningful indicators of impact in fragile settings, which ultimately highlights the need for tailored, context-responsive evaluation approaches.

The process of evaluating capacity building initiatives within FCAS therefore requires a departure from standardized static models toward process-oriented approaches that explicitly account for contextual volatility and operational constraints. While recognizing the importance of established evaluation strategies, there is a need for a consistent yet adaptable framework that supports evaluative decision-making across diverse contexts and modalities. By incorporating adaptive processes, such an approach can strengthen learning production, improve program design, and enhance effectiveness amid uncertainty and change. This aligns with calls for more adaptive evaluation approaches suited to complex and evolving settings, where learning and flexibility are prioritized over predefined approaches [11].

Taken together, and in response to the aforementioned, the Global Health Institute (GHI) at the American University of Beirut (AUB) in Lebanon developed a framework to guide the evaluation of GHCB programs in FCAS. Drawing on established theoretical models and five years of implementation and evaluation experience across multiple modalities, population groups, and periods of crisis in the MENA region, this paper presents a process-oriented evaluation framework for GHCB in FCAS aimed at understanding, refining, and strengthening program impact.

## Framework development process

Despite the proliferation of GHCB initiatives in the MENA region, evaluation of such programs, especially long-term evaluation, is scarce [12]. The proposed Evaluation of Capacity building (eCAP) framework addresses this gap by offering a comprehensive, culturally sensitive, adaptable, and scalable approach to evaluating GHCB in FCAS. Particularly, the development of eCAP is rooted in principles derived from Complex Adaptive Systems Theory, which center around non-linearity and continuous adaptation in dynamic environments. The eCAP framework (a process-oriented evaluation framework for GHCB in FCAS) has been developed through the evaluation of multiple capacity building initiatives implemented across the MENA region by the Academy Division under GHI [4] alongside critical examination of the strengths and limitations of established theoretical evaluation frameworks. This development methodology was employed to avoid reliance on single theoretical models or single-case evaluations. By combining systematic reviews with case study evaluations, this approach would enable the utilization of existing and established findings in the literature along with field-based and context-specific nuances that characterize fragile settings. It is designed to respond to diverse needs of populations living in FCAS and to accommodate the wide range of delivery modalities commonly employed in such contexts. By enabling a holistic understanding of capacity building outcomes across individual, organizational, and system levels, the framework provides practical guidance for evaluators and practitioners seeking to design, implement, and optimize evaluation strategies that strengthen health systems and improve learning outcomes in FCAS.

The eCAP framework was developed over a 5-year period, through iterative research, implementation, and refinement processes aimed at optimizing evaluation strategies for GHCB in FCAS as outlined below.

### Phase 1: situational assessment

Phase 1 involved a comprehensive situational assessment grounded in three systematic reviews that examined existing evidence on the evaluation and implementation of GHCB focusing on:

1. Methods used to evaluate global health capacity building initiatives in low- and middle-income countries [12]
2. Global health capacity building initiatives conducted in the Middle East and North Africa region [13]
3. Health research capacity building initiatives conducted in fragile and conflict-affected settings [14]

Details regarding synthesized findings of the 3 systematic reviews have been published elsewhere [4]. However, collectively, the reviews highlighted substantial heterogeneity in evaluation approaches, limited use of standardized tools, and an over-reliance on short-term outcome measures, with insufficient attention to sustainability, contextual adaptation, and longer-term impact. Evaluations were also predominantly oriented toward participant satisfaction with the program and immediate knowledge gains, largely neglecting longer-term outcomes related to sustainability, community-level outcomes, institutionalization, and system-level change.

This phase yielded key results that were synthesized into the following core outputs:

1. Development of standardized evaluation tools tailored to GHCB in FCAS.
2. Development of a standardized yet adaptable evaluation approach suitable for diverse delivery modalities, and populations, and levels of evaluation (individual, organizational, and community).
3. Strategic recommendations to integrate eCAP within GHI’s Academy to ensure alignment between implementation, learning, and evaluation processes. The Academy is the entity through which GHI delivers capacity building initiatives.

### Phase 2: case study production

Upon finalizing phase 1, the above-mentioned tools and planned approach were applied to evaluate a series of capacity building initiatives implemented by GHI’s Academy. These initiatives targeted several population groups, including health professionals, humanitarian workers, and vulnerable populations, each presenting with their own needs and challenges (see Box 1 for more details on each initiative).

**Box 1:** Example of population-sensitive evaluation: Mobile University for Health (MUH) [19]

The Mobile University for Health (MUH) program, implemented by the Global Health Institute at the American University of Beirut, targeted vulnerable women in Lebanon facing heightened health challenges due to ongoing crises and strained healthcare infrastructure. MUH aimed to empower these women as community health workers (CHWs) through capacity building and communities of practice (CoP)
During the evaluation, it was identified that the cultural context and background of the women participants played a crucial role. The program recognized that sensitive topics were met with discomfort when discussed in the presence of males. Therefore, the evaluation emphasized the need for gender-sensitive approaches in training and support for CHWs
*Implications for Evaluation and Future Initiatives:*
This example underscores the importance of sensitivity in the evaluation process. For future initiatives, especially in conflict-affected contexts, it highlights the necessity of considering and addressing concerns related to gender during program planning, implementation, and evaluation. Such insights contribute to the adaptability of evaluation strategies to the unique needs of the population

These GHCB initiatives (capacity building programs targeting health and humanitarian actors) were delivered through multiple modalities including in-person, online, and blended learning formats. This phase aimed to assess the effectiveness, feasibility, and contextual appropriateness of each modality, as well as their differential contributions to capacity development outcomes. This phase ultimately generated in-depth insights into the strengths and limitations of existing evaluation methodologies and implementation strategies, advancing the team’s understanding of how to meaningfully assess capacity building programs across different levels, populations, and delivery modalities.

Between 2020 and 2023, these evaluations culminated in the development and publication of 5 case studies reporting process and outcome-related evaluation results, and a conceptual paper, collectively serving as an empirical foundation for subsequent refinement of the eCAP framework [4, 15–19].

The aim was that, across these case studies, comparative attention would be given to how similarities and differences in key pillars of the respective evaluations (e.g., modalities used, populations targeted, evaluation levels addressed, etc.) shaped evaluation processes. Ultimately, this would enable the identification of common challenges, adaptive strategies, and subsequent considerations to inform the structure of the eCAP framework.

### Phase 3: framework development

**Fig. 1:**
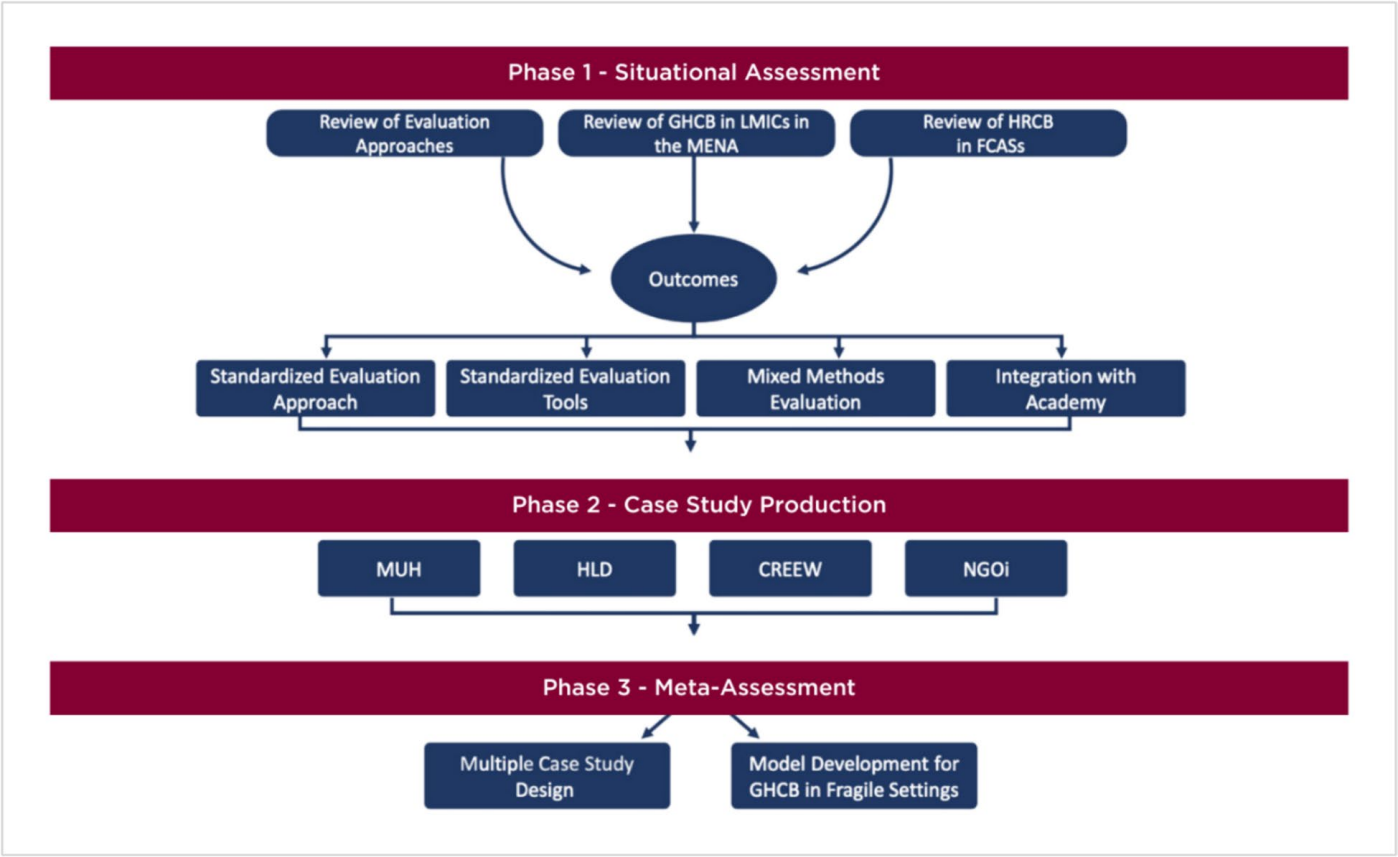
Development process of eCAP framework [4]

Phase 3 focused on the systematic synthesis of findings across all outputs in phases 1 and 2 to identify cross-cutting patterns, critical context-specific challenges, and success factors in evaluating capacity building initiatives (see Fig. 1). As opposed to relying on each initiative independently, lessons learned were therefore analyzed across population groups, delivery modalities, and levels of evaluation, and then distilled into a consolidated eCAP framework that integrates standardized evaluation components with flexibility around its application pathway. This process ultimately allowed the team to refine lessons learned and particularly to be mindful of how variations in the modality used, populations addressed, and contextual differences influence methodological decisions for the evaluation and its overall feasibility. The final framework, discussed in this manuscript, reflects the synthesis of empirical evidence from diverse fragile settings and is designed to support rigorous, context-sensitive evaluation of capacity building initiatives balancing robustness with practical feasibility in challenging environment.

Also, despite the fact that the evaluated GHCB initiatives differ in terms of content, modality, populations, and expected outcomes, conducting this comparative analysis supported the identification of a consistent set of pillars that are cross-cutting across the GHCB initiatives. These ultimately formed the analytical basis of the eCAP framework.

## Framework components

Key components of the eCAP framework were identified based on analytic comparisons of the GHCB initiatives evaluated, along with important insights generated from the situational analysis in phase 1 of the project. These components were produced based on recurring considerations identified from evaluations that were later grouped into broader domains.

**Fig. 2:**
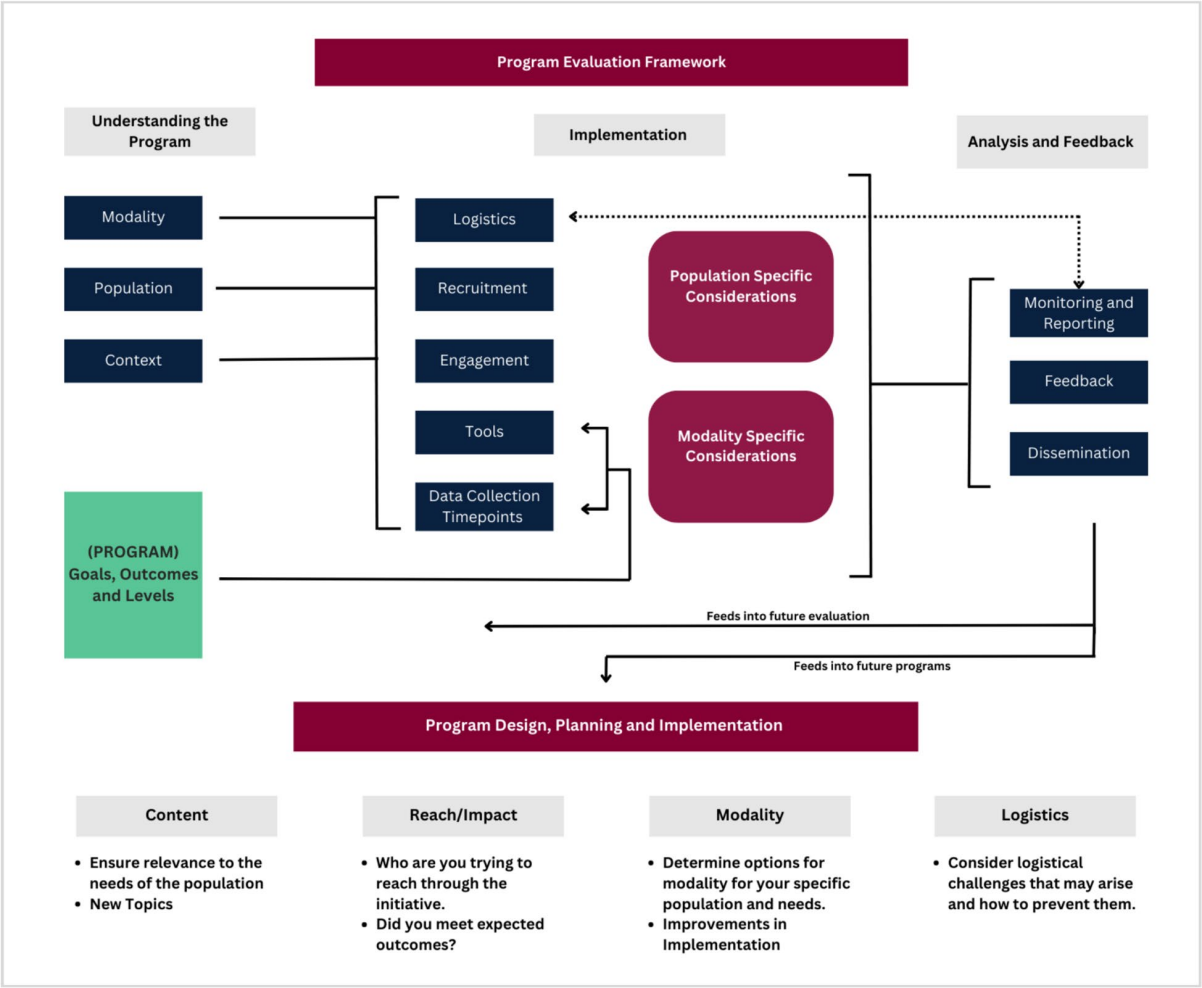
Final eCAP framework

The eCAP framework (see Fig. 2) guides evaluation of capacity building initiatives in FCAS, organized through three interconnected phases: (1) understanding the program, (2) implementation, and (3) analysis and feedback. The eCAP framework recognizes capacity building as a dynamic and context-dependent process, in which program design, delivery modalities, population characteristics, and contextual constraints directly influence evaluation design, tool selection, data collection methods, and overall feasibility. Rather than adopting a linear evaluation pathway, eCAP emphasizes adaptability and responsiveness to complexity, ensuring that evaluation process remains aligned with evolving realities.

### Understanding the program

This phase focuses on foundational planning elements that inform the design of a context-sensitive evaluation strategy. A clear understanding of the program’s structure, target population, delivery modality, and implementation context is essential to ensure that the evaluation approach is appropriate, meaningful, and feasible.

#### Modality

Identifying whether the capacity building initiative is delivered through online, in-person, or blended modality is a critical first step, as this directly shapes subsequent evaluation decisions. The choice of evaluation tools, timing of assessment, and modes of data collection are intrinsically linked to how learning is delivered and how participants engage with the program.

While online and blended modalities may expand reach by reducing physical access barriers, they often depend on reliable internet connectivity, which may be inconsistent in FCAS. In contrast, in-person delivery may increase engagement but is vulnerable to contextual disruptions. Importantly, evaluation approaches should align with the modality initially agreed upon by participants. For example, conducting in-person evaluations for a fully virtual program may introduce ethical and logistical challenges. Aligning evaluation methods with program modality therefore is likely to enhance accessibility and participant engagement.

#### Population

Similar to the above, understanding the characteristics and realities of the population targeted by a given capacity building initiative is essential for tailoring evaluation strategies (see Box 1). While modality and population are often interlinked, this relationship may not always be straightforward. For instance, online programs may implicitly target individuals with adequate digital literacy and access to technology, potentially excluding vulnerable groups who may be better reached through in-person modalities.

Population characteristics may also influence other essential factors such as the feasibility of longitudinal data collection, which is especially important to consider for long-term evaluations. Displaced, refugee, and vulnerable groups for instance often experience instability, mobility restrictions, and other challenges that may complicate or prevent long-term follow-up. In such cases, evaluations may prioritize short-term outcomes or employ mobile-friendly and flexible data collection approaches. In contrast, evaluations involving humanitarian workers or healthcare professionals may allow for longer-term follow-up, though participation may be constrained by workload and time limitations. Therefore, tailoring the evaluation approach to population-specific realities may minimize attrition and enhance engagement.

#### Context

A thorough understanding of implementation contexts is critical for designing resilient evaluation processes in FCAS. Such settings are characterized by political instability, security concerns, cultural sensitivities, and infrastructural limitations among others, which can limit participant engagement and data collection feasibility. Therefore, mapping and preparing for such challenges allow evaluators to develop contingency plans, including alternative data collection methods or adaptive timelines.

For example, across five years of delivering one of the Academy’s capacity building programs with community health workers in Lebanon, the research team encountered multiple contextual disruptions, including COVID-19 related lockdowns, fuel shortages, economic collapse, protests, civil unrest, and localized security threats among others. These realities were largely unforeseen, and had the potential to cause significant delays or to compromise many of the team’s initial evaluation plans. Accordingly, they necessitated rapid adaptation of evaluation plans, including shifting to remote data collection methods for example. Such experiences highlight the pivotal need for embedding flexibility and contextual awareness into evaluation designs to ensure continuity and limit inevitable disruptions.

#### Program logical framework

Although not directly part of the novelty presented in eCAP’s framework, ensuring clear articulation of program goals, outputs, and intended outcomes provides the foundation for effective framing of the evaluation. To that end, eCAP may be used in conjunction with logical frameworks traditionally used in humanitarian programming to align evaluation tools, indicators, and data collection timepoints with programmatic objectives. Ultimately, grounding the evaluation design through eCAP’s framework, and complementing it with clearly defined program logic ensure that findings are generated at appropriate moments, through adequate methods, and accurately reflect intended impacts.

### Implementation

Having finalized the planning process, the implementation phase focuses on operational considerations that may influence the feasibility and quality of evaluation activities throughout program delivery cycles.

#### Logistics

Logistical decisions are intricately tied to the understanding of contextual factors, including the uncertain and rapidly shifting sociopolitical conditions within fragile settings (see Box 2). Common challenges in such contexts include limited infrastructure, restricted mobility, and fluctuating security conditions. Developing contingency plans and maintaining adaptable evaluation tools capable of being administered in-person, online, or by phone, help ensure continuity of evaluation activities despite potential interruptions. Flexibility at this stage and contingency planning are essential to prevent delays, attrition, and data loss.

**Box 2:** illustrative example of logistical adaptation in evaluation: Mobile University for Health (MUH) Program [16]

Building on the MUH initiative, which empowered vulnerable women as community health workers, the program encountered unforeseen logistical challenges during the COVID-19 pandemic
Amidst pandemic constraints, the MUH program had to adapt its interview methods for CHWs due to COVID-19 safety measures. While phone interviews sufficed for individual interviews (SSIs), it became apparent that this approach was not effective for conducting focus group discussions (FGDs). This was due to technological challenges, unstable networks, and lack of experience in participating in group discussions remotely
In response, the program modified its approach. Recognizing the limitations of phone interviews for FGDs, MUH arranged a safe environment for in-person focus group discussions. This adaptation ensured meaningful participant engagement while adhering to health and safety guidelines
This example underlines the importance of logistical flexibility, especially in response to unforeseen circumstances like a global pandemic. It emphasizes the need for alternative methods and contingency plans, allowing the evaluation process to adapt to challenges while maintaining the integrity and effectiveness of data collection

#### Recruitment and engagement

Central to successful evaluation are effective recruitment and sustained participant engagement. To that end, tailored recruitment strategies are particularly important when working with hard-to-reach and vulnerable populations, especially when trust and accessibility may be limited. For example, collaborating with local organizations can enhance credibility, facilitate outreach, and support follow-up efforts for programs targeting vulnerable or displaced populations who may require additional trust.

Also, maintaining continuous engagement post-training is equally critical, particularly for long-term evaluations that utilize longitudinal data. This can include follow-up post-training and establishing and maintaining connections with organizations where evaluation may take place in the future. Accordingly, clearly communicating the value of participation and how feedback forms support programmatic improvements may increase response rates. It is important to treat participants as active contributors rather than passive recipients to strengthen the quality and authenticity of the data. This is especially important given the limited availability of long-term evaluation data in the region, which highlights the need for deliberate strategies to sustain engagement over time, including structured follow-up processes. The integration of technology can also serve as an essential element in enhancing participant interaction across various modalities during the evaluation process.

Moreover, engaging stakeholders who did not directly participate in the study requires previous contact and incentive, an example being organizational-level data collection whereby supervisors and peers may be contacted as part of a given evaluation. It is difficult to engage such stakeholders working within organizations when they have not been aware of, or directly part of, the capacity building initiative (see Box 3). Thus, communicating beforehand the importance of their contribution and the unique insights they can provide could increase the probability of them engaging in the evaluation process.

**Box 3:** Illustrative example of stakeholder engagement challenges in evaluation **Example: Humanitarian Leadership Diploma (HLD) Program **[15]

The Humanitarian Leadership Diploma (HLD) addressed the limited educational opportunities for humanitarian workers in the Middle East and North Africa (MENA) region, offering an online training program
During the evaluation of the HLD program, an attempt was made to gather organizational feedback through surveys sent to colleagues of the learners. However, a significant challenge emerged as there was a notably low response rate from these organizational surveys, which posed a considerable challenge. This limitation hindered the comprehensive understanding of organizational-level outcomes and insights into the program's impact beyond individual perspectives
To enhance the response rate for organizational surveys, future program evaluations could consider the following strategies:
• Clearly articulate the purpose of organizational surveys, emphasizing the value of feedback in improving program effectiveness
• Ensure that surveys are short and concise
• Implement follow-up communications to remind and encourage participation, emphasizing the collective benefit of organizational insights
*Implications for Future Program Evaluations:*
This example underscores the importance of anticipating and addressing challenges in gathering organizational feedback. Future initiatives should proactively plan for increased engagement in organizational surveys by incorporating effective communication strategies and optimizing survey design to encourage participation

#### Data collection tools

Selecting appropriate data collections is important to ensure alignment with program goals, as this is fundamental to capturing meaningful outcomes. Accordingly, quantitative tools may be well suited to measuring knowledge gains and immediate outcomes, while qualitative tools may allow for deeper exploration of experiences and perceptions, translation of knowledge into behaviors, and other key insights. To that end, mixed-methods approaches that triangulate both data sources are optimal to draw a more comprehensive assessment.

That said, tool accessibility and availability are particularly problematic in FCAS, as data collection tools are recommended to be linguistically appropriate, sensitive to literacy levels, and adaptable to multiple modes of delivery. As part of the eCAP framework, the team developed a standardized set of data collection tools to accompany the framework as a ready-for-use package, with the understanding that minor adjustments for contextual adaptation would be required to better fit the program, context, and related content. These include:

*Quantitative tools*:

- Knowledge Assessment, administered pre- and post-intervention to measure short-term knowledge gains aligned with program content.
- Course Evaluations administered post-intervention to assess learner satisfaction, content relevance, delivery quality, and facilitation, using a combination of scaled and open-ended questions.
- Community-Level Satisfaction Surveys to capture indirect effects of community-level capacity building initiatives on communities 3–6 months post-intervention by trained community health workers
- Organizational-Level Evaluation to assess long-term translation of knowledge of humanitarian workers/health professionals into organizational practices from the perspectives of colleagues, peers, and supervisors of participants 3–6 months post-intervention.

*Qualitative tools*:

- Focus Group Discussion Guide to explore community perceptions and indirect effects at community-level, including perceived behavioral changes of capacity building participants 3–6 months post-intervention
- Semi-Structured Interview Guide to examine learning experiences, knowledge application barriers and facilitators, and perceived behavioral changes 3–6 months post-intervention
- Reflective Commentary to elicit personal insights associated with the learning experience in writing, audio, or video formats.

#### Data collection timepoints

The timing of data collection periods is strategically related to intended outcomes and population characteristics. That is, immediate post-intervention assessments capture short-term learning, while delayed follow-ups capture long-term effects over time. Overall, in FCAS it is challenging to plan and implement long-term data collection efforts, especially for vulnerable and displaced groups who may show mobility constraints and other factors that lead to high attrition. Therefore, it is important to balance evaluation goals with realistic access considerations to increase the likelihood of capturing meaningful changes over time.

### Analysis and feedback

#### Monitoring and reporting

The importance of monitoring extends beyond immediate operational needs. It plays a pivotal role in the long-term success of capacity building initiatives by exploring patterns in implementation processes. By using the data collected over time, evaluators gain valuable insights into trends, emerging process issues and opportunities, and the evolution of program outcomes. This longitudinal perspective is instrumental in refining strategies, improving interventions, and enhancing the overall impact of capacity building initiatives. In fragile settings, where the context is prone to rapid changes, the ability to discern patterns through monitoring becomes particularly valuable for informed decision-making.

Moreover, ongoing monitoring and reporting mechanisms provide real-time analysis which allows evaluators to promptly identify challenges, implement corrective measures, and ensure the evaluation remains on course. This goes back to the logistics aspect of the framework, emphasizing the need for continues monitoring to identify potential challenges and manage them through previously established contingency plans.

#### Feedback

Structured and systematic feedback mechanisms are central to optimizing evaluation quality. This includes internal processes such as regular debriefing, monitoring, and lessons learned meetings, and external processes such as community validation processes where findings of a given evaluation are discussed with the targeted groups. These feedback systems are core components that should be embedded within institutions as routine practices to actively identify learnings, produce actionable recommendations, and ultimately improve programming.

## eCAP framework limitations

Despite the importance of this framework and its timeliness given the rapid advancement in the use of technology and innovative approaches in global health capacity building in fragile and conflict-affected settings, several limitations should be considered.

First, the framework is informed by experiences of evaluations conducted over a relatively medium term of 6 months, and it does not account for evaluations spanning longer periods of time. This is a crucial aspect, as it does not fully capture longer-term outcomes related to sustained behavioral change, institutionalization of learning, or system-level transformations. However, this decision was taken at the formative stages of the project due to the inherent challenges posed by conflict-affected settings such as instability, displacement, political vitality, shifting community dynamics among others, all of which complicate and potentially compromise data completeness if stretched over longer follow-up periods.

Second, the framework does not explicitly consider the wider impact evaluation at the level of systems, population health, and policies. While important in their own right, these dimensions were beyond the scope of the initiatives evaluated during the framework’s development. However, exploring the transfer of knowledge from the capacity building initiatives to systemic changes could provide valuable insights into the sustainability and scalability of such interventions, and this ultimately is a major limitation of the framework that could be further developed.

Finally, the framework is largely grounded in qualitative methodologies, aligning with its exploratory and process-oriented focus. While this is important to capture contextual nuances, the limited use of large-scale quantitative methods restricts generalizability. Accordingly, complementing this work with more elaborate quantitative approaches, especially those measuring long-term behavioral changes and involving larger samples, could strengthen subsequent evaluations using this framework.

## eCAP framework implications on research, practice, and policy

In conclusion, this paper presents a novel, process-oriented evaluation framework for GHCB in fragile and conflict-affected settings. The primary contribution of eCAP is in its field-based and empirically grounded development process that extends beyond static and linear models, and that ensures guidance for operations on key decision-making processes in evaluations. This includes selection of tools, recruitment of participants, management of logistics and others, based on pathways informed by lessons learned over a 5-year period and across multiple case studies. This is important because eCAP bridges between conceptual models for evaluations and the practical realities of implementing evaluations in fragile and conflict settings.

The eCAP framework has several important contributions across research, practice, and policy, particularly within FCAS.

*Implications for research*:

At the level of research, the eCAP framework addresses a critical gap in the evaluation of GHCB initiatives in FCAS, primarily in relation to its novelty being process-oriented and context-responsive. This ensures that evaluations are not merely outcome oriented and therefore account explicitly for implementation complexities in FCAS. This is especially important because the framework was developed through a lengthy iterative and systematic process that included conceptual outputs, systematic reviews, case studies, and evidence synthesis work over a 6-year time period. Particularly, this work advances understanding of how online, blended, and in-person learning approaches function under conditions of conflict and fragility.

*Implications for practice*:

From a practice perspective, the eCAP framework offers practitioners a practical, tested, and actionable roadmap for designing and implementing standardized yet adaptable evaluations in complex environments. In particular, its applicability across diverse modalities (online, blended, in-person), target populations (humanitarian workers, community health workers, health professionals), levels of evaluation (individual, community, and organizational), and fragile contexts enhances its utility.

*Implications for policy*:

At the policy level, the eCAP framework presents tools that may be used to produce evidence to inform funders, policymakers, and institutional stakeholders to support investments in evaluations to improve capacity building initiatives and the populations they serve. Accordingly, the framework supports better decision-making with regard to resource allocation and program design (Table 1).

**Table 1:** Description of capacity building initiatives under the academy

Initiative	Brief description	Population	Modality
Mobile University for Health	Builds professional health skills of displaced and host communities	Vulnerable displaced and host communities	Blended learning
Humanitarian Leadership Diploma	Equips humanitarian workers in the MENA region with relevant and contextualized leadership and technical skills to better manage humanitarian projects	Humanitarian workers	Online synchronous and online asynchronous
Center for Research and Education in the Ecology of War	Equips frontline health practitioners working in conflict settings with the necessary skills to conduct research on health and war	Frontline health practitioners	Blended learning
Non-Governmental Organizations Initiative	Enhances organizational capacities of organizations in the NGO sector	Civil society workers	In-person and online

## Data Availability

No datasets were generated or analyzed during the current study.
